# PRECIS-2: a tool to improve the applicability of randomised controlled trials

**DOI:** 10.1186/1745-6215-14-S1-O28

**Published:** 2013-11-29

**Authors:** Kirsty Loudon, Merrick Zwarenstein, Frank Sullivan, Peter Donnan, Shaun Treweek

**Affiliations:** 1 University of Dundee, Dundee, Scotland, UK; 2 Western University, London, Ontario, Canada; 3 University of Aberdeen, Aberdeen, Scotland, UK

## Background

RCTs are considered the most rigorous design to evaluate the effectiveness of different interventions but have generalizability issues. Our tool, PRECIS, could help trialists consider the effects of their design decisions on the applicability of their results in clinical settings.

## Aim

To produce an improved and validated version of PRECIS.

## Methods

Phase 1 involved brainstorming and a two-round Delphi survey of authors who cited PRECIS. Phase 2 involved discussion of the Delphi results by experienced trialists and alternative versions of PRECIS-2 developed and user-tested. Phase 3 will evaluate the validity and reliability of the most promising PRECIS-2 candidate using a sample of 15-20 trials rated by 15 other trialists.

## Results

Brainstorming sessions identified the PRECIS presentation (a wheel), lack of a scoring system and domain weighting as issues for exploration in the Delphi process. Thirty four completed responses from 90 invitees were received in Round 1 of the Delphi; Round 2 involved 23 individuals (response rate 82%). 45% selected a 1-5 Likert scale, 56.5% wanted to use a table (to justify decisions) *and* a PRECIS wheel, 26% were in favour of weighting domains. Suggestions for extra domains included: recruitment process for participants and integration of the intervention into the healthcare system. An expert panel in Toronto used the Delphi suggestions to help create alternative versions of PRECIS-2 for user-testing in spring 2013.

## Conclusions

PRECIS can be improved by the addition of a Likert scale and additional domains. We expect to have a validated PRECIS-2 by the beginning of 2014.

